# The Impact of Fillers on UV-Aging of Rotomolded Polyethylene Items: A Case Study on Ignimbrite Dust, *Arundo donax* L. Fibers, and Their Combination

**DOI:** 10.3390/ma18204723

**Published:** 2025-10-15

**Authors:** Francisco Romero, Jake Kelly-Walley, Mark McCourt, Luis Suárez, Zaida Ortega

**Affiliations:** 1 Departamento de Ingeniería de Procesos, Universidad de Las Palmas de Gran Canaria, 35017 Las Palmas de Gran Canaria, Spain; francisco.romero@ulpgc.es; 2 Matrix Polymers, Unit 2, Compass Industrial Park, Spindus Road, Speke, Liverpool L24 1YA, UK; jake.kelly-walley@matrixpolymers.com; 3 School of Mechanical and Aerospace Engineering, Queen’s University Belfast, Ashby Building, Stranmillis Road, Belfast BT9 5AH, UK; 4 Sustainable Polymers and Composites Divisions, AMIC, Queen’s University of Belfast, Ashby Building, Belfast BT9 5AH, UK; m.mccourt@qub.ac.uk; 5 Departamento de Ingeniería Mecánica, Universidad de Las Palmas de Gran Canaria, 35017 Las Palmas de Gran Canaria, Spain; luis.suarez@ulpgc.es

**Keywords:** rotational molding, composite, UV-ageing, carbonyl index, tensile properties

## Abstract

Different composites of polyethylene and two fillers (ignimbrite dust and *Arundo donax* fibers) were obtained by rotational molding. Both fillers were also combined among them to produce hybrid composites. The blends, prepared by dry-blending, were later rotomolded to determine the effect of such fillers into the tensile properties of the materials, before and after subjecting them to accelerated weathering on a UV chamber for up to 500 h. No significant differences are observed in the mechanical behavior of the different sample series, regardless their type or ratio of filler (5 or 10% by weight), due to the modifications only taking place on the sample surface and the rotomolded items having a thickness of nominally 4 mm. The carbonyl index was obtained from the FTIR spectra, determining an increase in this parameter with irradiation time. The samples with the *Arundo* fibers exhibit a lower carbonyl index, showing the potential stabilization effect of this lignocellulosic filler against UV, while the composites with the mineral powder tend to increase the oxidation of the samples when included at high loadings (10%).

## 1. Introduction

Rotational molding, or rotomolding, is a versatile manufacturing process for producing seamless, hollow parts, mainly starting from polymer powders. During this process, the polymer is introduced into the mold, which is then heated and rotated in two axes, allowing the powder to evenly coat the interior surfaces of the mold. After the polymer sinters and melts, the mold is cooled down, solidifying the product for its removal [1,2,3,4]. This technique is particularly advantageous for fabricating large components with complex geometries and provides a cost-effective solution with reduced material wastage compared to conventional methods like injection molding [3,5,6]. The key strengths of rotational molding include its ability to create stress-free parts with uniform wall thicknesses and the absence of significant pressure during processing, which minimizes the risk of defects such as voids or warping [7,8,9]. Additionally, the simplicity of mold design and lower costs associated with the production of rotomolded components makes it accessible to a broad range of applications, from automotive parts to playground equipment, leisure and sports, or outdoor furniture [3,6,7,10,11].

Despite the strengths outlined for rotomolding, it does still present certain limitations. One significant drawback is the longer cycle times required for heating and cooling, which can hinder production efficiency and affect cost-effectiveness for high-volume manufacturing [9,12]. Additionally, the nature of the materials used can lead to increased brittleness in the final product, particularly when blends or fillers are incorporated [1,12]. In the quest for sustainability, researchers and manufacturers are exploring several paths to enhance the environmental credentials of rotational molding. Innovations include the development of biodegradable composites from natural fibers, such as lignocellulosic materials, which not only reduce dependency on fossil fuels but also facilitate biodegradability [12,13,14]. The integration of recycled materials and waste from other industrial processes also serves the dual purpose of minimizing resource consumption and closing the loop in material life cycles. In this sense, some research has also been performed on the use of recycled polymers into the process, particularly using post-consumer plastics, although with certain limitations derived from the particularities of the process [15,16,17,18,19]. Moreover, side-streams from the food or wood industry, such as wheat bran, banana fibers, maple dust, or giant reed, among others, have been introduced into rotomolded items [1,11,20,21,22,23,24,25,26], while also inorganic/mineral dust from various origins, such as glass, stone or residues from metal extraction [27,28,29,30,31,32,33], have been studied. Finally, some other developments to advance towards sustainability are the optimization of heating times and mold designs, together with the use of robotic arms, to shorten cycle durations and improve energy efficiency [30,34,35,36,37].

The long cycle times of the rotomolding process can result in oxidation of the polymer, therefore needing of incorporating stabilizers into their formulation. Therefore, the incorporation of stabilizers and UV-protective agents in rotomolded polymers is crucial to counter potential degradation mechanisms that can occur due to thermal and UV-exposure [38,39,40,41,42]. This degradation can lead to significant losses in mechanical properties, dimensional integrity, and overall performance of the rotomolded products [43]. Although polyethylene is generally considered stable against photo-oxidative degradation due to its [CH_2_–CH_2_]_n_ structure, certain internal and external factors, including catalyst residues from synthesis or the presence of the fillers may increase its susceptibility to oxidation. The formation of oxidized groups can further accelerate degradation, as they enhance UV absorption within the polymer matrix [44].

Thermal stabilizers, such as hindered amine light stabilizers (HALSs), are known to effectively combat photo-oxidative degradation in polymers like PE and PP during processing. Research indicates that HALSs combined with other UV absorbers can significantly enhance the longevity and durability of rotomolded composites by slowing the rate of oxidative degradation initiated by UV light exposure [45,46,47]. Moreover, crosslinking agents such as dicumyl peroxide (DCP) contribute to thermal stability by reducing mobility within the polymer chains, thus improving insulation properties. This stability is especially beneficial during the extended heat treatment phases of rotomolding [41,45]. The incorporation of bioactive fillers into rotomolded items represents a significant area of research in materials science, particularly in recent times, due to the unique processing characteristics and potential applications of rotomolding technology. In this sense, biobased materials from waste streams, such as black tea and coffee, are promising fillers for rotomolded composites due to their potential for enhanced functional properties, including antioxidant activities. For instance, composites with 5 wt.% coffee silverskin maintained comparable tensile strength to unmodified PE, indicating that such biowaste-derived fillers could reinforce while also providing other beneficial properties [21,48]. The antioxidative capabilities attributed to both spent coffee grounds and cacao husks improve the OIT within the polymer matrix, therefore indicating that plant-based fillers are not merely physical reinforcements, but they can also confer significant chemical resistance to degradation associated with UV-exposure and thermal aging. A different research work conducted by Aniśko and Barczewski explored the application of LDPE filled with black tea waste, who found that black tea waste affects the thermal and mechanical behavior of the composites, contributing to their oxidative stability [49]. Similarly, other lignocellulosic fillers, such as those derived from *Arundo donax* or *Solidago canadiensis*, exhibit high antioxidants content, which can be transferred to the PE matrix for enhanced OIT and UV-stability [50,51,52]. In conclusion, introducing bioactive fillers into rotomolded products not only enhances material properties but also aligns with sustainability goals by utilizing waste materials. Ongoing research should continue to focus on optimizing filler types and processing conditions to fully exploit their potential in functional composites. Finally, the modifications in the melt flow characteristics of the PE occurred as a consequence of the additives should not be disregarded, as they directly affect the processing and final properties of rotomolded items [48].

This point is relatively unexplored, particularly for rotomolded items, this being the main focus of this communication. This is particularly critical as this degradation can result in a loss of mechanical properties and structural failures in applications exposed to outdoor conditions.

Balancing the amount and type of filler is crucial to achieving optimal performance in rotomolded PE items while maintaining adequate UV resistance. Different degradation behaviors have been observed for distinct natural fillers [53,54]; lignin is a crucial component to absorb the UV radiation and reduce the photodegradation as it absorbs the radiation and protects the cellulose, keeping the structural integrity of the composite, which means that lignin acts as a degradation inhibitor [55]. The preparation of nanocomposites can also significantly enhance resistance to UV radiation. For instance, the use of diatomaceous earth combined with UV stabilizers has shown potential for developing active packaging, which could also mitigate UV effects in rotomolded products [56]. The degradation rates associated with UV-exposure, especially when combined with humid environments, can be effectively reduced through the application of properly selected additives [52,57,58]. Similarly, mesoporous silica has demonstrated enhanced UV protection along with good thermal stability, which suggests potential applications in the rotomolded polymer industry where durability and stability under UV-exposure are paramount [47,59]. Furthermore, the implementation of biodegradable and sustainable UV filters in polymer formulations provides an innovative approach to enhancing environmental stability while reducing overall degradation from harmful radiation [60].

Therefore, a careful selection and optimization of the additives introduced into rotomolded products can result in composite materials that better withstand the rigors of outdoor exposure, thereby extending the lifespan of the products into which they are incorporated. In this work, ignimbrite dust (a volcanic rock mainly based on silica and aluminum and iron oxides) and giant reed (*Arundo donax* L.) fibers have been incorporated into PE-based composites, as a promising avenue for enhancing material performance while addressing environmental sustainability, as they both come from waste streams. Ignimbrite dust is associated with high thermal stability and mechanical reinforcement [27]. On the other hand, giant reed fibers have been proof as an effective renewable reinforcement material due to their inherent mechanical properties and their rapid growth [61]. Both fillers have been dry-blended with the PE matrix, producing composites with a single filler and also hybrid composites, from the combination of both fillers together at the same proportion, focusing on how tensile properties evolve with exposure to UV radiation. Testing these composites after varying durations of UV-exposure can provide important insights into their durability and longevity in outdoor applications, as laboratory UV-weathering chambers can accelerate the degradation process and predict the alteration of materials when subjected to outdoor conditions [54,55,62,63].

## 2. Materials and Methods

### 2.1. Materials

The material used was a polyethylene—hexene grade from Matrix Polymers (Liverpool, UK) (Revolve N-307), with a density of 0.939 g/cm^3^ and a melt flow index of 3.5 g/10 min. The reed fibers were obtained following a chemo-mechanical procedure, consisting in soaking the stems of the plant into a NaOH solution for 1 week and later subjecting the vegetal material to a series of rollers to remove the soft materials and release the fibers [61]; fibers exhibit an approximate length of 3–4 mm, with diameters of 150 μm [61]. The ignimbrite dust was supplied by Compañía Artesanal de Cantería de Arucas S.L. (Arucas, Spain) (density of 2.45 g/cm^3^ and particle size between 6–50 μm) and was used without any modification [27].

### 2.2. Preparation of Composites

The composites were prepared by dry-blending the different materials into the adequate masses to obtain 5 and 10% composites by weight, as generally higher loadings result in processability problems and high proportion of voids. For hybrid composites, equal amounts of fiber and dust were used. The different blends prepared are shown in Table 1.

The blends prepared were introduced into a 200 × 200 × 200 mm aluminum mold with a vent hole in a biaxial three-arm carrousel rotomolding machine from Ferry RotoSpeed (Ferry Industries, Stow, OH, USA), RS–1600 Turret Style model to obtain the rotomolded test samples. The rotational speed ratio was set to 8:2, and the oven temperature was set at 300 °C. The oven and peak internal air temperature (PIAT) were constantly monitored using a Rotolog system until reaching a IAT of 180 °C. The cooling was performed with forced air until reaching 70 °C, when parts were extracted from the mold. The total weight of material in each sample was 800 g, which allowed obtaining ≈4 mm-thick parts.

### 2.3. Characterization of Samples

#### 2.3.1. Rheology Assessment—Flow Behavior of Composites

The flow properties of the materials were determined at 190 °C in an oscillatory rheometer AR G2 from TA Instruments, using parallel plates geometry of 25 mm diameter, with a gap of 1.5 mm, under a nitrogen atmosphere. Preliminary assays were performed under the strain sweep mode in order to ensure that frequency and flow tests were performed under the linear viscoelastic region (LVR). In these tests, the strain was varied between 0.1 and 5%. Frequency sweep tests were performed at 0.5% strain (in the LVR), in the 100 to 0.01 Hz range. Finally, flow tests were also performed at the same temperature, between 0.01 and 1 Hz shear rate.

#### 2.3.2. Tensile Testing and UV-Weathering

Once the different series were rotomolded, standard test samples were cut to dumbbell shape suitable for tensile testing (170 mm length, 10 mm wide, ≈4 mm thickness), producing 5 samples for each one. They were then introduced into a UV-weathering chamber Q-Sun Xenon (Q-Lab, Baltimore, MD, USA), where the dumbbell-shaped samples were subjected to UV radiation at 340 nm and energy irradiation of 0.89 W/m^2^, at a temperature of 65 °C for different periods of time, ranging from 100 to 500 h. Samples were later characterized to determine their mechanical behavior, following an in-house protocol at Matrix Polymers, consisting in a tensile test in a LRX-device from Lloyd Instruments (Bognor Regis, UK), at 50 mm/min. Results are shown as average values of minimum 3 replicates, including standard deviations.

#### 2.3.3. Carbonyl Index Determination by Fourier Infrared Spectroscopy

Fourier Infrared spectroscopy (FTIR) was employed to assess the level of oxidation of the samples by determining the carbonyl index (CI) of each sample. The spectra were analyzed with a particular emphasis in the regions of 1850–1700 and 1500–1420 cm^−1^, corresponding to the carbonyl and C-H bending, taken as reference, as it is distinguishable and measurable in all samples [64]. The CI was calculated by dividing the area under these peaks, presenting results as average values of the different spectra collected (3 per sample). The calculation also was done by dividing the maxima (absorption) of the peaks within this range. Spectra were obtained in a Perkin Elmer Spectrum Two device (Waltham, MA, USA) in the ATR mode, in the range between 4500 and 700 cm^−1^, at a resolution of 4 cm^−1^, and 64 accumulations.

## 3. Results and Discussion

### 3.1. Cycle Time Analysis

**Figure 1:**
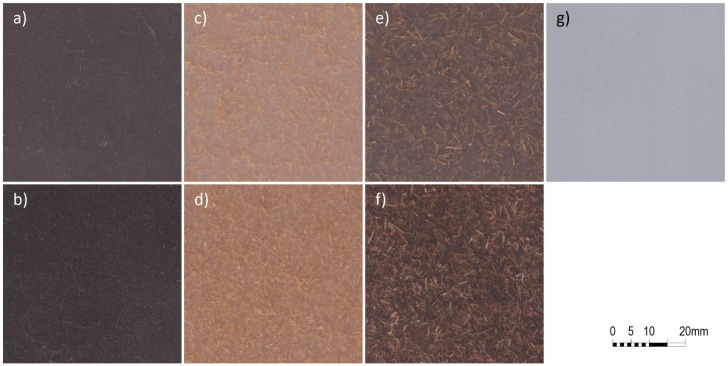
Rotomolded samples for (**a**) 5% ignimbrite dust, (**b**) 10% ignimbrite dust, (**c**) 5% *Arundo* fibers, (**d**) 10% *Arundo* fibers, (**e**) 5% hybrid composites: 2.5% *Arundo* fibers and 2.5% ignimbrite dust, (**f**) 10% hybrid composites: 5% *Arundo* fibers and 5% ignimbrite dust, and (**g**) neat PE.

All rotomolded parts show a good consistency, with low number of voids and good filler distribution, both for the fibers and the dust (Figure 1). The composites, particularly those with reed fibers, do not show any unpleasant odor, indicative of fiber thermal degradation.

Table 2 summarizes the reductions in heating, cooling, and total process cycles for the different moldings:

For composites with reed fibers it is observed that oven time tends to increase with the fiber content, particularly on oven time. Previous works have shown that composites with abaca fiber also increase cycle time [24]. On the opposite, ignimbrite composites are obtained with a light reduction of cycle time, as also explained in a previous work of authors [30]. The changes in oven time are mainly due to the modifications in the induction time, reduced for ignimbrite composites as a consequence of the lower heat capacity of the mineral, and increased for lignocellulose fillers due to their thermal insulating properties. Hybrid composites show an intermediate behavior between the fibers and particles ones, although it seems that the influence of the lignocellulose is higher than that of the ignimbrite.

### 3.2. Rheology Assessment of Rotomolded Composites

The rheological behavior of the materials plays an essential role in rotomolding. High viscosities would impede an appropriate flow of the material inside the mold and hinder the migration of air bubbles in the densification step, while a low viscosity would result in inappropriate consolidation of the parts [65,66].

**Figure 2:**
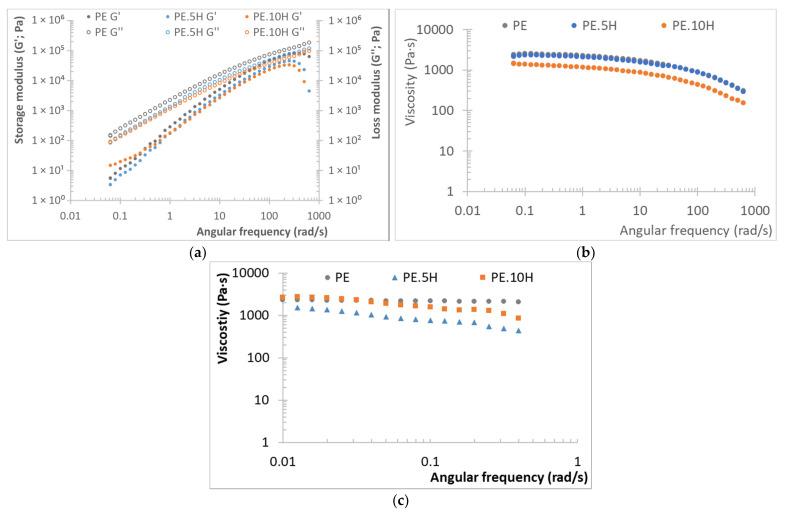
Rheological behavior of PE hybrid rotomolded samples: (**a**) storage (left) and loss (right) modulus versus angular frequency, (**b**) viscosity versus angular frequency, (**c**) viscosity at very low angular frequencies.

As observed in Figure 2, the rheological behavior of hybrid composites is very close to that of the neat PE. Hybrid composites show no significant alterations on storage or loss modules (Figure 2a), as also happening for ignimbrite and *Arundo* composites (separately introduced in the matrix), where only storage modulus is slightly increased for composites at 10%, particularly at low frequencies, with loss modulus remains overlapped for all materials [30,67]. This is translated in all the samples series showing similar viscoelastic behavior, with a well-defined Newtonian plateau, and similar viscosity values along the entire range of shear rates studied (Figure 2b), with all samples exhibiting a certain shear thinning behavior at high shear rates; in fact, it is clearly seen that the viscosity curves for neat PE and hybrid composites at 5% are overlapped for the entire range of analysis. This means that the incorporation of any of the fillers, or their combination, affects the polymer flow properties; this is demonstrated by the excellent processability of the different blends, which are molded without any difficulty and allows for the obtaining of parts with good quality, as already mentioned. Viscosity at very low angular frequencies is also very similar to that of the neat matrix, with more differences for 5% hybrid composites than for 10% ones, but still within the same range of magnitude (Figure 2c); the neat PE exhibits a less pronounced shear-thinning behavior than the composites, which might be related to a weakening of the interactions among the PE chains due to the incorporation of the fillers. As observed in Figure 2a, the storage modulus of neat PE is generally higher than that of its composite counterparts, although the differences are almost negligible, indicating a stronger material response to deformation in these regions [68]. This phenomenon is attributed to the enhanced interfacial stress transfer between the polyethylene matrix and any filler; the presence of filler particles and fibers disrupts the cohesive interactions among PE chains, resulting in a more fluid-like behavior under mechanical stress, as observed by the slightly reduced viscosity (more pronounced for higher ratios of fillers). Consequently, while pristine PE maintains a stable structural network, the composites experience disruptions in their network due to filler interactions, which can lead to a decrease in modulus as the filler content increases [68,69].

Generally, the incorporation of fillers tends to produce an increase in viscosity [28,49], while some fillers (basalt dust) have shown a behavior close to that reported here [70], that is, no effect on the viscous behavior of the matrix. The reduced effect of the fillers in the rheological behavior might be due to the relatively low ratio of fillers used. Finally, the zero-shear viscosity values (η_0_) show a similar behavior for all the sample series, therefore concluding that the fillers do not have an effect on this parameter (2300 Pa·s for neat PE, and maximum differences for hybrid composites, with 1672 Pa·s for PE.5H and 2862 Pa·s for PE.10H).

### 3.3. Tensile Properties

**Figure 3:**
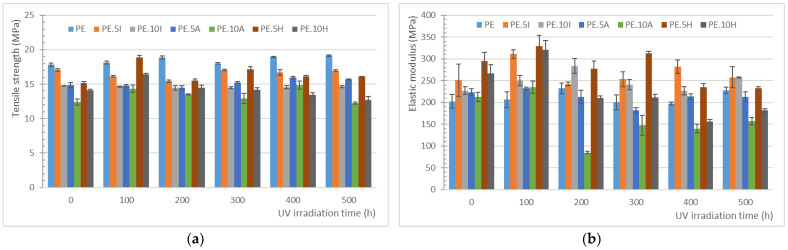
(**a**) Evolution of tensile strength for the different composites along the 500 h of UV-irradiation, (**b**) variation of elastic modulus for the different composites through the UV-irradiation.

The tensile testing of the different sample series before the UV-irradiation shows the expected trend, namely, a slight reduction in tensile strength, more significant with increased filler content, with an increase in elastic modulus (Figure 3). As also expected, elongation at break is significantly reduced with the incorporation of the fillers, passing from over 250% for the neat PE to less than 20% for all composites, related to an increased brittleness brought by the low ductility and higher rigidity of the fillers. This reduced ductility can bring the advantage of a higher dimensional stability. Elongation at break for all composites show no significant difference among the different series, being only significant (*p* < 0.001) for comparisons with neat PE.

The properties of polyethylene remain mainly steady after the irradiation, with no significant variations in any of the properties measured (*p*-value for strength: 0.150, for tensile modulus: 0.536, and for elongation at break: 0.997). However, a slight trend towards a reduction in the elongation at break can be observed, together with a light growing trend in the elastic modulus and tensile strength, which is related to a minimum increase in the stiffness of the material, which marks the beginning of the degradation of the neat PE matrix. It was expected to obtain a reduction in the elongation at break for samples, due to the embrittlement of the matrix induced by the UV-radiation, as found in previous works [71,72]. In semi-crystalline polymers, such as PE, such embrittlement is primarily attributed to a decrease in molar mass caused by a chain scission process. Literature proposes a series of events as a mechanism for this degradation: chain scission leads to molar mass reduction, which induces chemicrystallization, followed by a decrease in interlamellar spacing, ultimately resulting in embrittlement. On the other hand, since molecular entanglements facilitate plastic deformation under tensile loading [72,73], the disruption of this entanglement network due to chain scission impedes plastic deformation, making the material brittle.

In the context of UV-irradiation, these degradation mechanisms do not occur uniformly across the material’s thickness. The lack of significant differences in the mechanical behavior of the different series might be related to the samples thicknesses (of nominally 4 mm); most works in the literature analyze the effects of UV-irradiation on sheets or films, where the UV can affect most of the material, while in this case only surface is attacked. For instance, the work carried out by Barczewski et al. [50], who proposed the use of *Solidago canadensis* as a stabilizer of a PE matrix, in sheets of 2 mm thick. For samples subjected to 100 h of UV-irradiation, they found a slight increase of elastic modulus and tensile strength, although already got a significant reduction in elongation at break, more significant for the samples of neat PE, demonstrating the stabilization effect of certain fractions of that plant filler. Hsueh et al. found lower differences in the mechanical behavior [72] on samples of nominally 3.24 mm thick; an assessment of micromechanical properties through the sample thickness, up to 600 μm depth, revealed an increased superficial modulus, that is, there is an increased stiffness on the superficial layers of the samples as a result of the limited oxidation induced by UV and air contact. The highest differences in this work were observed also when working with sheets, although authors do not specify their thickness. The work by López-Martínez et al. is based on 25 μm-thick films, which also explains the higher variation in the results obtained after the UV-exposure [44]. Similarly, a research conducted by Gulmine et al. reports the variations in carbonyl index depth profiles indicate that oxidation proceeds heterogeneously in HDPE [74]. This non-uniform degradation leads to spatial differences in mechanical properties through the thickness, increasing the likelihood of overall brittle failure. Therefore, the results obtained in this work are not directly comparable to those in the literature, due to the different conditions of the testing. In any case, the trends found towards the reduction of elongation at break and increase in modulus follow the expected behavior, although not being significant from the statistic point of view, as already mentioned. On the development of this research work, some other assays, such as thermogravimetric analysis and differential scanning calorimetry were performed, but no differences were found due to the UV-exposure due to the bulk of the material remaining unchanged because of the samples thickness.

It can also be observed that the composites provide a distinct behavior along the exposure time to the UV-irradiation. For all composites series, elastic modulus remains unchanged for the entire test and tensile strength is only affected for the hybrid composites. Elongation at break is only modified (tendency to increase) for composites with ignimbrite. Table 3 summarizes the results from the ANOVA analysis of results:

For PE.5I the most significant difference arises in tensile strength for samples after 200 h UV-exposure, which is lowered. In elongation at break, however, significant differences are observed between the unexposed samples and the exposed ones, regardless the time of the UV-exposure. The maximum *p*-value found for this comparison is 0.13. The differences, despite not significant, decrease with the exposure time, that is, there is a bigger difference between the samples exposed for 100 h and 500 h than for those exposed for 400 and 500 h, which somehow shows that the higher the exposure the higher the brittleness, as otherwise expected. On the other hand, the series with 10% ignimbrite dust also shows significant differences between series, although only between unexposed samples and samples after 500 h, and samples exposed 100 and 500 h. This could be related to a certain protective effect of the mineral at higher loadings against the radiation.

For hybrid composites a different trend is observed, with higher variations in modulus, not significant yet, with significant differences in tensile strength in three groups: unexposed, samples exposed for 100 h, and remaining expositions times. Tensile strength increases about a 15% for both samples series, and then is reduced by the same proportion for the samples over 300 h, which could be related to a higher extent of the degradation.

### 3.4. Carbonyl Index: Samples Surface Oxidation

**Figure 4:**
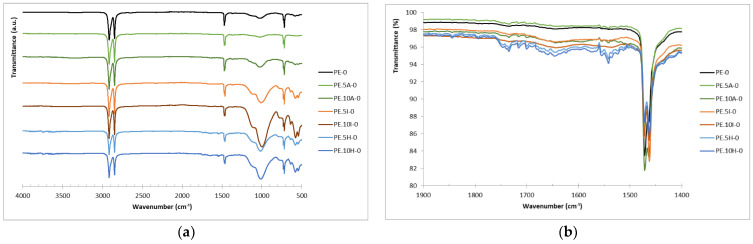

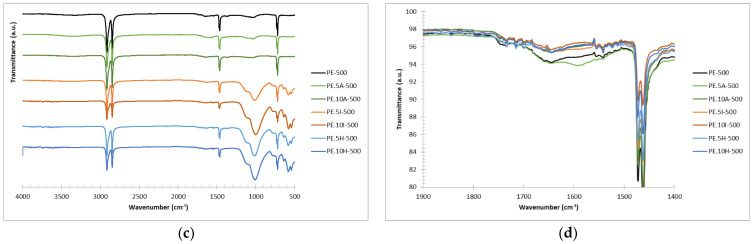
FTIR spectra for the different samples series: (**a**) comparison of the spectra obtained for the different samples before the UV-exposure, (**b**) closer observation of the area of interest for the carbonyl index (1800 to 1400 cm^−1^), (**c**) comparison of the different materials after 500 h of UV-exposure, (**d**) area of interest for CI for samples exposed for 500 h.

For all FTIR spectra (Figure 4), characteristic absorption bands of polyethylene are evident at 2850 and 2915 cm^−1^, corresponding to the CH_2_ stretching vibrations in the polymer chains. Additionally, bands at 1469 cm^−1^ associated with the deformation and bending vibrations of CH_2_ and CH_3_ groups are also observed. For composites, particularly for those containing the reed fibers, a broad band (not too intense) is observed at 3200–2600 cm^−1^, attributed to hydroxyl groups in lignocellulose, together with a higher intensity of the band at 1200–900 cm^−1^, typical for C=O bonds in cellulosic compounds (Figure 4a) [75]. In counteract, the composites with ignimbrite dust exhibit a more pronounced absorption band at around 1000 cm^−1^, related to the Si-O-Si bonds in the mineral [27].

The determination of carbonyl index was performed in two ways, namely, using the areas under the peaks mentioned (carbonyl, at about 1720 cm^−1^, and reference peak, at around 1450 cm^−1^) and the peak heights, observing similar trends for both parameters. The calculations performed on the area are considered more representative, as they consider different species containing carbonyl groups. For instance, the research conducted by López-Martínez et al. [44] shows notable changes in the spectra of UV-aged samples after 144 and 288 h, especially at 1718 cm^−1^ and 1600 cm^−1^, which are attributed to the formation of carbonyl-containing functional groups. The increased intensity and broadening of the carbonyl band suggest the presence of various photo-oxidation products such as ketones, esters, and anhydrides. Similarly, Almond et al. [64] propose the determination of carbonyl index using the area under the spectra and not only the peak heights due to the complexity of polyolefin degradation, which results in the formation of different carbonyl compound, such as ketones at 1714 cm^−1^, γ-lactones (1780 cm^−1^), esters and/or aldehydes (1733 cm^−1^), and carboxylic acids (1700 cm^−1^). Therefore, measuring the total area under the carbonyl region provides a more comprehensive and reliable indicator of oxidative degradation. To evaluate the progression of degradation over time, the carbonyl index was calculated for the different composites at the different times of UV-exposure. Figure 4c shows the full spectra for the different materials after 500 h of exposure, where the increased band at about 1700 cm^−1^ can be seen as an inflection in the base line, being the observation clearer in Figure 4d, where the region of interest for the CI is presented. The increased oxidation can be clearly seen in this figure, where the base line in this region has almost disappeared and is now made of different absorption bands, corresponding to different species.

Table 4 shows the average values (and standard deviation) for carbonyl index of the different sample series at the different exposure times. In general terms, it can be observed that the longer exposure times result in a higher CI; similarly, research conducted by López-Martínez et al. indicated a marked increase in the carbonyl index of low-density polyethylene films after exposure to UV radiation [44], even finding a direct correlation between weathering time and the carbonyl index, indicating progressive oxidative degradation of the polymer matrix. The formation of carbonyl groups is indicative of chain scission and oxidation processes occurring within the material. In this sense, Russo et al. reported on the photodegradation effects on greenhouse films composed of PE, noting an increase in viscosity and gel content, both of which are indicative of crosslinking and degradation phenomena [76]. The increase in carbonyl content in these films confirmed the polymer’s response to UV-exposure, supporting the notion that the degradation impacts both mechanical properties and molecular structure.

**Figure 5:**
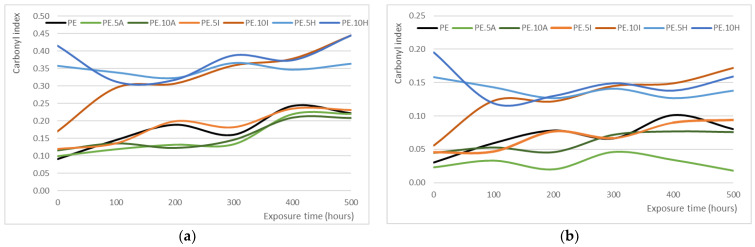
Evolution of carbonyl index along time for the different series. The graph has been represented with lines for a better observation of the trends mentioned. (**a**) CI calculated from areas, (**b**) CI calculated from peak heights.

For the series of materials included within this research work, the trends are similar to those described in the literature. The starting point (i.e., carbonyl index for unexposed samples) is similar for *Arundo* composites, and slightly higher for ignimbrite series, being higher with the mineral content. The CI for hybrid composites samples is remarkably higher, which might be due to the interaction between the fibers and the ignimbrite dust or the presence of fibers closer to the samples surface (Figure 4b also shows the higher relevance of these bands in unexposed samples). However, for these samples series, the CI remains steady along the 500 h of the experiment, with a slight reduction in these parameters for intermediate times, which are attributed rather to the small sections taken in the reading rather to actually distinct oxidation status; it therefore seems that these composites are not negatively affected by the radiation, possibly related to the certain protective effect lignocellulose material. For neat PE, the oxidation induced has statistical significance from 200 h (*p*-values of <0.001 for CI calculated both by areas and heights, respectively), meaning that the material is stable up to 100 h of UV-exposure (Figure 5). Carbonyl index is higher when calculated from areas, as already explained due to the consideration of more species than merely carboxylic acids, although the trend observed is similar for both parameters, regardless their calculation method.

**Figure 6:**
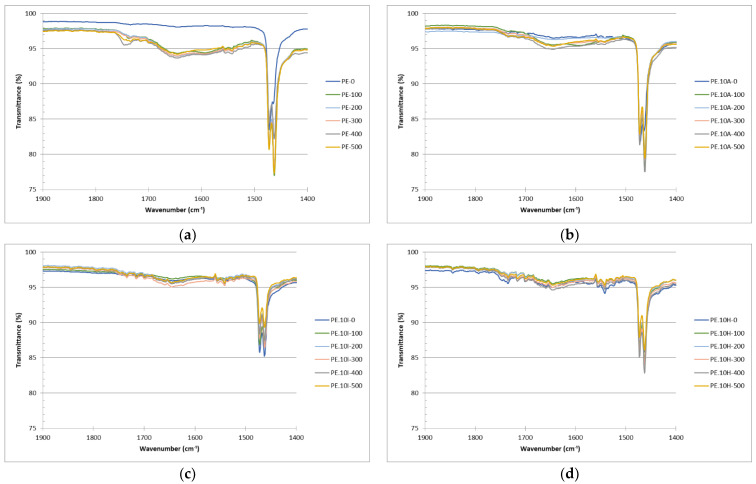
Evolution of FTIR spectra for the different UV-exposure periods: (**a**) neat PE, (**b**) PE.10A, (**c**) PE.10I, (**d**) PE.10H.

Even if the differences for PE are considered with statistical significance from 200 h, the spectra show a very distinct behavior for the samples after only 100 h (Figure 6a). Unexposed PE samples show an essentially flat curve in the 1800–1600 cm^−1^ region, which means that no carbonyl groups are detected, while with the UV-exposure some new bands at about 1720–1740 cm^−1^, related to ketones, which are the dominant carbonyl species produced in the photooxidation of PE [77]. A shoulder at higher wavelengths, generally assigned to aldehydes/esters, is also observed for samples after higher exposure times. The lack of evident bands at about 1600 cm^−1^ suggests that carboxylate salts are not forming, possibly due to the effect of stabilizers in the bulk, which is consistent with the no (or very minor) band at approximately 1710 cm^−1^, related to hydrogen-bonded acids or conjugated carbonyls, which only appears when oxidation is very advanced, which is not the case for the samples studied in this work, and which are more likely due to acetylacetone (ketone).

For composites, as already explained, there are lower differences along time, particularly for composites with the lignocellulosic filler. For instance, composites with giant reed show similar level of transmittance to neat PE in this range of wavelengths only for samples after 400 h, with the samples exposed for shorter times almost showing a flat profile, being this trend more evident for PE.10A series (Figure 6b). The composites with ignimbrite show still less relevance of these bands (Figure 6c), with obtaining in fact similar CI than composites with reed, while hybrid materials show the wide absorption band in this region which explains the higher CI, even for unexposed samples (Figure 6d).

For composites containing reed fibers no significant differences are observed with exposure time until reaching 400 h, showing the stabilizing effect of the *Arundo* into the matrix. The CI remains lower for 100, 200 and 300 h of exposure, reaching similar levels to that of neat PE for longer times (still lower in any case). The effect is more pronounced for the series with 10% of fibers, showing a positive effect of the filler content. Another work using carbon black and carbon nanotubes found that during the initial exposure period (0–192 h), the films containing carbon structures exhibited similar trends in carbonyl index, with those incorporating graphene and carbon black showing lower values than the control film without additives. This suggests that these structures help inhibit carbonyl group formation under UV radiation. At extended exposure times (192–480 h), films containing 2 wt.% multi-walled carbon nanotubes demonstrated the lowest carbonyl index, indicating superior UV protection compared to graphene and CB. The similar behavior of graphene- and CB-containing films in terms of oxidized structure formation suggests a comparable degree of UV shielding between these two additives [44]. Similarly, the work by Barczewski et al. using *Solidago canadiensis* [50] has shown the potential of certain sections of plant in the stabilization of a PE matrix, although the analysis was only performed for 100 h in that case.

For ignimbrite composites, the ratio of dust has a more significant effect. For 5% composites, a certain protective effect is found for up to 100 h; in fact, three different groups are obtained (consistent for both parameters): 0 and 100 h, 200 and 300, and 400 and 500 h. For the entire times range, this series shows the same behavior than neat PE, that is, the ignimbrite dust is not providing any effect on the material, nor reducing neither accelerating the oxidation of the matrix. However, for 10% composites the carbonyl index is always higher than for neat PE, which might reflect that the mineral dust might be concentrating the radiation rather than dispersing it. Some other authors have reported that the incorporation of clays into PE matrices result in a faster photodegradation of the polymer, while some other compounds not based on silica but on zinc oxide or iron ore are able to decrease the speed and extent of the degradation [78,79]. No FTIR tests were conducted on those samples though, which were thinner than the samples used in this work, as already mentioned; calcium carbonate is thought to reduce the photooxidation of PE, although no explanations or mechanisms are proposed in that work [80]. The role of silica-based materials on the oxidative degradation of polyethylene samples under UV-exposure appears to be still unclear; while some authors have shown their potential to accelerate oxidation processes via pro-oxidant activity in some studies [81], other works suggest that it might also have a protective role that mitigate the photooxidation [59]. The results provided in this work add some light on a potential protective effect of the ignimbrite dust on the PE matrix, as per the reduced carbonyl index for earlier stages of UV-exposure.

## 4. Conclusions

The effect of bio and silica-based materials on polyethylene matrices regarding the UV stability is not fully addressed in literature yet. The results obtained within this work show a certain protective effect of the inorganic filler (ignimbrite dust, a by-product of quarries), based on the carbonyl index calculations, particularly for 5% loadings. Further research should be conducted to state this point in any case. Giant reed derived materials show a more significant reduction of the carbonyl index compared to neat PE after the exposure to the UV lighting, which is related to their phenolic content, which acts protecting the PE from the photooxidation. However, these materials are more sensitive to moisture than those containing the mineral dust, which might have a significant effect on their durability when subjected to outdoor exposition. The hybrid composites show an intermediate behavior, being more affected in their mechanical properties than the series with just the ignimbrite or the reed, although also showing a more stable carbonyl index, with FTIR spectra unchanged for the full experiment.

Rheological tests show no significant differences of the composites compared to the neat polyethylene, which is in line with the excellent moldability and good aesthetics of all sample series.

The interaction between the fillers should be studied more in detail, and the production of films or thinner sheets might be useful to have a better understanding on how the weathering is affecting the material bulk. In this research work, due to the samples thickness not many differences in the behavior of the different sample series were observed (mechanical or thermal analysis); working with thinner samples would allow for establishing a more accurate effect of the fillers within the bulk material. In this sense, a separate analysis considering moisture uptake and its effects on the polymer should also be performed in order to have a better understanding on the behavior of such materials under actual outdoor conditions. The research should also focus on establishing the mechanism for the degradation of the PE and the potential changes due to the fillers.

## Figures and Tables

**Table 1:** Composites prepared by dry-blending in this work (% *w*/*w*).

Short Name	PE	Ignimbrite	Giant Reed
PE	100	-	-
PE.5A	95	-	5
PE.10A	90	-	10
PE.5I	95	5	-
PE.10I	90	10	-
PE.5H	95	2.5	2.5
PE.10H	90	5	5

**Table 2:** Extension of each stage of the rotomolding cycle and variations compared to the neat PE molding cycle.

Cycle	Oven Time		Cooling Time		Total Cycle Time	
	(min)	Variation (%)	(min)	Variation (%)	(min)	Variation (%)
PE	10.7	--	27.6	--	38.3	--
PE.5A	11.6	8.4	27.5	−0.4	39.1	2.1
PE.10A	12.8	19.6	27.9	1.1	40.7	6.3
PE.5I	10.5	−1.9	27.0	−2.2	37.5	−2.1
PE.10I	9.7	−9.3	26.5	−4.0	36.2	−5.5
PE.5H	11.1	3.7	27.7	0.4	38.8	1.3
PE.10H	12.0	12.1	27.3	−1.1	39.3	2.6

**Table 3:** Summary of the statistical analysis of significance (*p*-values) for the sample series along the UV-exposure.

Samples Series	Modulus	Tensile Strength	Elongation at Break
PE	0.536	0.150	0.997
PE.5A	0.279	0.138	0.089
PE.10A	0.081	0.907	0.215
PE.5I	0.596	0.098	0.001
PE.10I	0.161	0.968	0.006
PE.5H	0.077	0.036	0.135
PE.10H	0.061	0.046	0.559

**Table 4:** Summary of the carbonyl index (calculated by area and peak height).

Material	Exposure Time (h)	CI (Area)	CI (Heights)
PE	0	0.090 ± 0.010	0.030 ± 0.001
	100	0.145 ± 0.036	0.059 ± 0.013
	200	0.189 ± 0.040	0.078 ± 0.021
	300	0.160 ± 0.016	0.066 ± 0.014
	400	0.243 ± 0.029	0.101 ± 0.011
	500	0.222 ± 0.006	0.080 ± 0.015
PE.5A	0	0.099 ± 0.023	0.039 ± 0.006
	100	0.119 ± 0.033	0.042 ± 0.009
	200	0.132 ± 0.020	0.051 ± 0.008
	300	0.133 ± 0.046	0.053 ± 0.014
	400	0.219 ± 0.034	0.088 ± 0.012
	500	0.220 ± 0.018	0.086 ± 0.003
PE.10A	0	0.116 ± 0.000	0.045 ± 0.000
	100	0.136 ± 0.008	0.053 ± 0.005
	200	0.123 ± 0.019	0.046 ± 0.011
	300	0.146 ± 0.011	0.072 ± 0.013
	400	0.209 ± 0.040	0.077 ± 0.010
	500	0.208 ± 0.004	0.076 ± 0.000
PE.5I	0	0.120 ± 0.003	0.046 ± 0.001
	100	0.136 ± 0.007	0.047 ± 0.002
	200	0.199 ± 0.015	0.077 ± 0.005
	300	0.182 ± 0.016	0.067 ± 0.006
	400	0.235 ± 0.001	0.090 ± 0.000
	500	0.231 ± 0.001	0.094 ± 0.001
PE.10I	0	0.170 ± 0.013	0.056 ± 0.005
	100	0.295 ± 0.029	0.123 ± 0.008
	200	0.307 ± 0.021	0.122 ± 0.005
	300	0.359 ± 0.031	0.145 ± 0.003
	400	0.378 ± 0.025	0.149 ± 0.003
	500	0.445 ± 0.003	0.172 ± 0.007
PE.5H	0	0.358 ± 0.013	0.158 ± 0.010
	100	0.339 ± 0.016	0.143 ± 0.001
	200	0.323 ± 0.013	0.127 ± 0.006
	300	0.366 ± 0.022	0.141 ± 0.016
	400	0.347 ± 0.001	0.127 ± 0.005
	500	0.364 ± 0.018	0.138 ± 0.013
PE.10H	0	0.416 ± 0.050	0.195 ± 0.027
	100	0.313 ± 0.010	0.119 ± 0.006
	200	0.318 ± 0.002	0.130 ± 0.005
	300	0.388 ± 0.013	0.149 ± 0.011
	400	0.374 ± 0.012	0.138 ± 0.002
	500	0.445 ± 0.023	0.159 ± 0.009

## Data Availability

The original contributions presented in this study are included in the article. Further inquiries can be directed to the corresponding author.

## References

[1] Hejna A., Barczewski M., Andrzejewski J., Kosmela P., Piasecki A., Szostak M., Kuang T. (2020). Rotational Molding of Linear Low-Density Polyethylene Composites Filled with Wheat Bran. Polymers.

[2] Ghanem Z., Šourkova H.J., Sezemský J., Špatenka P. (2022). The Effect of Plasma Treatment of Polyethylene Powder and Glass Fibers on Selected Properties of Their Composites Prepared via Rotational Molding. Polymers.

[3] Kelly-Walley J., Martin P., Ortega Z., Pick L., McCourt M. (2024). Recent Advancements towards Sustainability in Rotomoulding. Materials.

[4] Ghanem Z., Sasidharan S.P., Jenikova Z., Špatenka P. (2019). Rotational Molding of Plasma Treated Polyethylene/Short Glass Fiber Composites. Int. J. Eng. Manag. Sci..

[5] Ortega Z., McCourt M., Romero F., Suárez L., Cunningham E. (2022). Recent Developments in Inorganic Composites in Rotational Molding. Polymers.

[6] Guan C.F., Peng W., Qin L., Zhang Y.C., Yang W.M. (2013). A Numerical Investigation on Temperature Field in the Oven of a Rotational Molding Machine. Key Eng. Mater..

[7] Gupta N., Ramkumar P., Sangani V. (2020). An Approach toward Augmenting Materials, Additives, Processability and Parameterization in Rotational Molding: A Review. Mater. Manuf. Process..

[8] Hanana F.E., Rodrigue D. (2015). Rotational Molding of Polymer Composites Reinforced with Natural Fibers. Plast. Eng..

[9] Hanana F.E., Rodrigue D. (2018). Rotational Molding of Self-Hybrid Composites Based on Linear Low-Density Polyethylene and Maple Fibers. Polym. Compos..

[10] Gupta N., Ramkumar P. (2021). Effect of Industrially Processed Glass Fibre Dust on Mechanical, Thermal and Morphological Properties Mixed with LLDPE for Rotational Molding Process. Sadhana—Acad. Proc. Eng. Sci..

[11] Kelly-Walley J., Ortega Z., McCourt M., Millar B., Suárez L., Martin P. (2023). Mechanical Performance of Rotationally Molded Multilayer MLDPE/Banana-Fiber Composites. Materials.

[12] Greco A., Maffezzoli A. (2015). Rotational Molding of Biodegradable Composites Obtained with PLA Reinforced by the Wooden Backbone of Opuntia Ficus Indica Cladodes. J. Appl. Polym. Sci..

[13] Cisneros-López E.O., Pérez-Fonseca A.A., González-García Y., Ramírez-Arreola D.E., González-Núñez R., Rodrigue D., Robledo-Ortíz J.R. (2018). Polylactic Acid-Agave Fiber Biocomposites Produced by Rotational Molding: A Comparative Study with Compression Molding. Adv. Polym. Technol..

[14] Aniśko J., Barczewski M., Mietliński P., Piasecki A., Szulc J. (2022). Valorization of Disposable Polylactide (PLA) Cups by Rotational Molding Technology: The Influence of Pre-Processing Grinding and Thermal Treatment. Polym. Test..

[15] Cestari S.P., Martin P., Hanna P., Kearns M., Mendes L.C. (2021). Rotational-Moulded Building Blocks for the Circular Economy. Mater. Sci. Forum.

[16] Shaker R., Rodrigue D. (2019). Rotomolding of Thermoplastic Elastomers Based on Low-Density Polyethylene and Recycled Natural Rubber. Appl. Sci..

[17] Dou Y., Rodrigue D. (2022). Morphological, Thermal and Mechanical Properties of Recycled HDPE Foams via Rotational Molding. J. Cell. Plast..

[18] Pick L., Hanna P.R., Gorman L. (2022). Assessment of Processibility and Properties of Raw Post-Consumer Waste Polyethylene in the Rotational Moulding Process. J. Polym. Eng..

[19] Díaz S., Ortega Z., McCourt M., Kearns M.P., Benítez A.N. (2018). Recycling of Polymeric Fraction of Cable Waste by Rotational Moulding. Waste Manag..

[20] Hanana F.E., Rodrigue D. (2021). Effect of Particle Size, Fiber Content, and Surface Treatment on the Mechanical Properties of Maple-Reinforced LLDPE Produced by Rotational Molding. Polym. Polym. Compos..

[21] Hejna A., Barczewski M., Kosmela P., Mysiukiewicz O., Kuzmin A. (2021). Coffee Silverskin as a Multifunctional Waste Filler for High-Density Polyethylene Green Composites. J. Compos. Sci..

[22] Ortega Z., Monzón M.D., Benítez A.N., Kearns M., McCourt M., Hornsby P.R. (2013). Banana and Abaca Fiber-Reinforced Plastic Composites Obtained by Rotational Molding Process. Mater. Manuf. Process..

[23] Cisneros-López E.O., González-López M.E., Pérez-Fonseca A.A., González-Núñez R., Rodrigue D., Robledo-Ortíz J.R. (2017). Effect of Fiber Content and Surface Treatment on the Mechanical Properties of Natural Fiber Composites Produced by Rotomolding. Compos. Interfaces.

[24] Ortega Z., Suárez L., Kelly-Walley J., McCourt M. (2023). Mechanical Properties of Rotomolded Parts with Abaca Fiber: Effect of Manufacturing with 1, 2 or 3 Layers. Compos. Theory Pract..

[25] Suárez L., Ortega Z., Romero F., Paz R., Marrero M.D. (2022). Influence of Giant Reed Fibers on Mechanical, Thermal, and Disintegration Behavior of Rotomolded PLA and PE Composites. J. Polym. Environ..

[26] León L.D.V.E., Escocio V.A., Visconte L.L.Y., Junior J.C.J., Pacheco E.B.A.V. (2020). Rotomolding and Polyethylene Composites with Rotomolded Lignocellulosic Materials: A Review. J. Reinf. Plast. Compos..

[27] Romero F., Suárez L., Díaz S., Ortega Z. (2025). Rotomolded Polypropylene—Ignimbrite Composites: Giving a Second Life to Mineral Dust Wastes. Polym. Compos..

[28] Barczewski M., Hejna A., Aniśko J., Andrzejewski J., Piasecki A., Mysiukiewicz O., Bąk M., Gapiński B., Ortega Z. (2022). Rotational Molding of Polylactide (PLA) Composites Filled with Copper Slag as a Waste Filler from Metallurgical Industry. Polym. Test..

[29] Gupta N., Ramkumar P. (2021). Rheological and Thermal Investigation of Industrially Processed Glass Fiber Blended with Linear Low-Density Polyethylene for Rotational Molding Process. Trans. Indian Inst. Met..

[30] Ortega Z., Douglas P., Hanna P.R., Kelly-Walley J., McCourt M. (2024). Influence of Mold Pressurization on Cycle Time in Rotational Molding Composites with Welded Ignimbrite as Loading. Compos. Commun..

[31] Aniśko J., Bartczak D., Barczewski M. (2023). Limitations of Short Basalt Fibers Use as an Effective Reinforcement of Polyethylene Composites in Rotational Molding Technology. Adv. Sci. Technol. Res. J..

[32] Głogowska K., Pączkowski P., Samujło B. (2022). Study on the Properties and Structure of Rotationally Moulded Linear Low-Density Polyethylene Filled with Quartz Flour. Materials.

[33] Ghanbarpour B., Moslemi A. (2022). Evaluation of Mechanical, Optical, and Antibacterial Properties of Metal—Oxide Dispersed HDPE Nanocomposites Processed by Rotational Molding. Polym. Compos..

[34] Qin L., Ding Y.M., Jiao Z.W., Liu Y.X., Yang W.M. (2013). The Research on the Heating Time of Rotational Molding. Key Eng. Mater..

[35] Luciano G., Vignolo M., Brunengo E., Utzeri R., Stagnaro P. (2023). Study of Microwave-Active Composite Materials to Improve the Polyethylene Rotomolding Process. Polymers.

[36] McCourt M., Kearns M.P., Martin P., Butterfield J. A Comparison between Conventional and Robotic Rotational Moulding Machines. Proceedings of the IMC34—34th International Manufacturing Conference.

[37] Malnati P. (2019). New Era for Rotomolding? Innovative Technology and Equipment Offer Superior Process Control and Produce Better Parts with Higher Repeatability and Reproducibility. Plast. Eng..

[38] Azwa Z.N., Yousif B.F., Manalo A.C., Karunasena W. (2013). A Review on the Degradability of Polymeric Composites Based on Natural Fibres. Mater. Des..

[39] Moraczewski K., Stepczyńska M., Malinowski R., Karasiewicz T., Jagodziński B., Rytlewski P. (2019). The Effect of Accelerated Aging on Polylactide Containing Plant Extracts. Polymers.

[40] Dopico-García M.S., Castro-López M.M., López-Vilariño J.M., González-Rodríguez M.V., Valentão P., Andrade P.B., García-Garabal S., Abad M.J. (2011). Natural Extracts as Potential Source of Antioxidants to Stabilize Polyolefins. J. Appl. Polym. Sci..

[41] Ahmad H., Rostami-Tapeh-Esmaeil E., Rodrigue D. (2024). The Effect of Chemical Crosslinking on the Properties of Rotomolded High Density Polyethylene. J. Appl. Polym. Sci..

[42] Quiles-Carrillo L., Montanes N., Jorda-Vilaplana A., Balart R., Torres-Giner S. (2019). A Comparative Study on the Effect of Different Reactive Compatibilizers on Injection-Molded Pieces of Bio-Based High-Density Polyethylene/Polylactide Blends. J. Appl. Polym. Sci..

[43] Weizman O., Uziel A., Mead J., Dodiuk H., Ophir A., Kenig S. (2022). Quantitative Analysis of UV Protective Additives in Polyethylene Films by Solvent Extraction Coupled with UV Spectrophotometry. Polym. Adv. Technol..

[44] López-Martínez E.D., Martínez-Colunga J.G., Ramírez-Vargas E., Sanchez-Valdes S., Ramos-de Valle L.F., Benavides-Cantu R., Rodríguez-Gonzalez J.A., Mata-Padilla J.M., Cruz-Delgado V.J., Borjas-Ramos J.J. (2022). Influence of Carbon Structures on the Properties and Photodegradation of LDPE/LLDPE Films. Polym. Adv. Technol..

[45] Ahmad H., Rodrigue D. (2025). Development of Lightweight and Thermally Insulative Crosslinked Polypropylene via Rotomolding. Polym. Eng. Sci..

[46] Liu C., Mei C., Xu B., Chen W., Yong C., Wang K., Wu Q. (2018). Light Stabilizers Added to the Shell of Co-Extruded Wood/High-Density Polyethylene Composites to Improve Mechanical and Anti-UV Ageing Properties. R. Soc. Open Sci..

[47] Barczewski M., Aniśko-Michalak J., Skórczewska K., Maniak M., Kosmela P., Żukowska W., Przybylska-Balcerek A., Szwajkowska-Michałek L., Stuper-Szablewska K., Waliszewska B. (2025). Correlation between Rotational Molding Process Temperature and Degradation Changes of Polyethylene and Composites Containing Coffee Spent Grains Used as an Active Filler. Sustain. Mater. Technol..

[48] Hejna A., Barczewski M., Kosmela P., Mysiukiewicz O. (2023). Comparative Analysis of the Coffee and Cocoa Industry By-Products on the Performance of Polyethylene-Based Composites. Waste Biomass Valorization.

[49] Aniśko J., Barczewski M. (2023). Uniaxial Rotational Molding of Bio-Based Low-Density Polyethylene Filled with Black Tea Waste. Materials.

[50] Barczewski M., Aniśko J., Suárez L., Skórczewska K., Rackov M., Ortega Z., Hejna A. (2024). Using the Potential of Canadian Goldenrod (*Solidago Canadensis*) as a Functional Filler with Antioxidant Activity of Low-Density Polyethylene Composites as an Example of Sustainable Development of an Invasive Plant. Ind. Crops Prod..

[51] Suárez L., Ortega Z., Barczewski M., Cunningham E. (2023). Use of Giant Reed (*Arundo donax* L.) for Polymer Composites Obtaining: A Mapping Review. Cellulose.

[52] Díaz S., Romero F., Suárez L., Tcharkhtchi A., Ortega Z. (2025). A Preliminary Study on the Use of Microalgae Biomass as a Polyolefin Stabilizer. Iran. Polym. J. (Engl. Ed.).

[53] Mahzan S., Fitri M., Zaleha M. (2017). UV Radiation Effect towards Mechanical Properties of Natural Fibre Reinforced Composite Material: A Review. IOP Conf. Ser. Mater. Sci. Eng..

[54] Nasri K., Toubal L., Loranger É., Koffi D. (2022). Influence of UV Irradiation on Mechanical Properties and Drop-Weight Impact Performance of Polypropylene Biocomposites Reinforced with Short Flax and Pine Fibers. Compos. Part C Open Access.

[55] Peng Y., Liu R., Cao J. (2015). Characterization of Surface Chemistry and Crystallization Behavior of Polypropylene Composites Reinforced with Wood Flour, Cellulose, and Lignin during Accelerated Weathering. Appl. Surf. Sci..

[56] Charoensri K., Shin Y.J., Park H.J. (2023). Innovative HDPE Composites Enriched with UV Stabilizer and Diatomaceous Earth/Zinc Oxide for Enhanced Seafood Packaging and Antimicrobial Properties. Polymers.

[57] Christmann J., Gardette J.-L., Pichon G., Bouchut B., Therias S. (2021). Photostabilization of Polyethylene by a Hindered Amine Light Stabilizer in Blooming Conditions and Impact of MDO Processing. Polym. Degrad. Stab..

[58] Takacs K., Tatraaljai D., Pregi E., Huszthy P., Pukanszky B. (2022). Synthesis and Evaluation of a Novel Natural-Based Phosphine Antioxidant for the Thermal Stabilization of Polyethylene. J. Therm. Anal. Calorim..

[59] Lee S.J., Lee Y.J., Lee J.Y., Yun Y.S., Kwon T.K., Jung I. (2025). Development of Biocompatible Mesoporous Silica Materials for Enhanced UV Protection. Bull. Korean Chem. Soc..

[60] Nizamov R., Poskela A., Kaschuk J., Henn K.A., Grande R., Granroth S., Nyberg M., Esmaeilzadeh M., Vapaavuori J., Österberg M. (2025). Sustainable Nanocellulose UV Filters for Photovoltaic Applications: Comparison of Red Onion (*Allium cepa*) Extract, Iron Ions, and Colloidal Lignin. ACS Appl. Opt. Mater..

[61] Suárez L., Barczewski M., Kosmela P., Marrero M.D., Ortega Z. (2023). Giant Reed (*Arundo donax* L.) Fiber Extraction and Characterization for Its Use in Polymer Composites. J. Nat. Fibers.

[62] Beg M.D.H., Pickering K.L. (2008). Accelerated Weathering of Unbleached and Bleached Kraft Wood Fibre Reinforced Polypropylene Composites. Polym. Degrad. Stab..

[63] Chang B.P., Mohanty A.K., Misra M. (2020). Studies on Durability of Sustainable Biobased Composites: A Review. RSC Adv..

[64] Almond J., Sugumaar P., Wenzel M.N., Hill G., Wallis C. (2020). Determination of the Carbonyl Index of Polyethylene and Polypropylene Using Specified Area under Band Methodology with ATR-FTIR Spectroscopy. e-Polymers.

[65] Greco A., Maffezzoli A., Forleo S. (2014). Rotational Molding of Bio-Polymers. AIP Conf. Proc..

[66] Bellehumeur C.T., Bisaria M.K., Vlachopoulos J. (1996). An Experimental Study and Model Assessment of Polymer Sintering. Polym. Eng. Sci..

[67] Ortega Z., Suárez L., Kelly-Walley J., Hanna P.R., McCourt M., Millar B. (2024). Use of Pressure in Rotational Molding to Reduce Cycle Times: Comparison of the Thermomechanical Behavior of Rotomolded Reed/Polyethylene Composites. J. Compos. Sci..

[68] Yoon S.W., Lee S., Choi I.S., Do Y., Park S. (2015). Electrical and Mechanical Properties of Polyethylene/MWCNT Composites Produced by Polymerization Using Cp2ZrCl2 Supported on MWCNTs. Macromol. Res..

[69] Pandey A.K., Singh K., Kar K.K. (2017). Thermo-Mechanical Properties of Graphite-Reinforced High-Density Polyethylene Composites and Its Structure–Property Corelationship. J. Compos. Mater..

[70] Barczewski M., Mysiukiewicz O., Andrzejewski J., Piasecki A., Strzemięcka B., Adamek G. (2021). The Inhibiting Effect of Basalt Powder on Crystallization Behavior and the Structure-Property Relationship of α-Nucleated Polypropylene Composites. Polym. Test..

[71] Jabarin S.A., Lofgren E.A. (1994). Photooxidative Effects on Properties and Structure of High—Density Polyethylene. J. Appl. Polym. Sci..

[72] Hsueh H.-C., Kim J.H., Orski S., Fairbrother A., Jacobs D., Perry L., Hunston D., White C., Sung L. (2020). Micro and Macroscopic Mechanical Behaviors of High-Density Polyethylene under UV Irradiation and Temperature. Polym. Degrad. Stab..

[73] Hiss R., Hobeika S., Lynn C., Strobl G. (1999). Network Stretching, Slip Processes, and Fragmentation of Crystallites during Uniaxial Drawing of Polyethylene and Related Copolymers. A Comparative Study. Macromolecules.

[74] Gulmine J.V., Janissek P.R., Heise H.M., Akcelrud L. (2003). Degradation Profile of Polyethylene after Artificial Accelerated Weathering. Polym. Degrad. Stab..

[75] Suárez L., Hanna P.R., Ortega Z., Barczewski M., Kosmela P., Millar B., Cunningham E. (2024). Influence of Giant Reed (*Arundo donax* L.) Culms Processing Procedure on Physicochemical, Rheological, and Thermomechanical Properties of Polyethylene Composites. J. Nat. Fibers.

[76] Russo P., Acierno D., Marinucci L., Greco A., Frigione M. (2013). Influence of Natural and Accelerated Weathering on Performances of Photoselective Greenhouse Films. J. Appl. Polym. Sci..

[77] Celina M.C., Linde E., Martinez E. (2021). Carbonyl Identification and Quantification Uncertainties for Oxidative Polymer Degradation. Polym. Degrad. Stab..

[78] Patwary F., Mittal V. (2015). Degradable Polyethylene Nanocomposites with Silica, Silicate and Thermally Reduced Graphene Using Oxo-Degradable Pro-Oxidant. Heliyon.

[79] de Morais F.L.D., Medeiros F.d.S., Silva G.G., Rabello M.S., de Sousa A.R. (2017). Photodegradation of UHMWPE Filled with Iron Ore Fine. Mater. Res..

[80] Van Nguyen K., Nguyen T.T., Nguyen D.T., Pham H.T.T. (2018). Effect of CaCO_3_ Filler on the Degradation of High Density Polyethylene (HDPE) Film Containing Prooxidants. Vietnam. J. Sci. Technol..

[81] Redhwi H.H., Siddiqui M.N., Andrady A.L., Syed H. (2013). Durability of LDPE Nanocomposites with Clay, Silica, and Zinc Oxide II. Weatherability of the Nanocomposites. Polym. Compos..

